# The Impact of Health Insurance Policy on the Health of the Senior Floating Population—Evidence from China

**DOI:** 10.3390/ijerph15102159

**Published:** 2018-10-01

**Authors:** Yingying Meng, Junqiang Han, Siqi Qin

**Affiliations:** 1 Centre for Social Security Studies, Wuhan University, Wuhan 430072, China; yingyingmeng@whu.edu.cn; 2 School of Public Management, South-Central University for Nationalities, Wuhan 430074, China; 3 Department of Sociology, The Chinese University of Hong Kong, Shatin 999077, NT, Hong Kong; 1155076790@link.cuhk.edu.hk

**Keywords:** medical insurance system, health, adverse selection, floating population, China

## Abstract

The impact of health insurance on residents’ health is one of the focal points of academic research. Due to the fact that China’s medical insurance system is composed of a variety of programs and that the pooling districts are at the lower administrative level, enrollment in different medical insurance programs or at different places may have certain influences on the health of residents. This has mostly been neglected by previous studies. This paper uses data from the 2015 China Migrants Dynamic Survey (CMDS), focusing on the senior floating population and taking the difference in government subsidy proportions as an instrumental variable in order to identify the effects of health insurance programs and regional differences on the health of the senior floating population. Three effects were observed: First, participation in the health insurance system significantly improves floating seniors’ self-rated health. Second, the health status of floating seniors affects their choice of health insurance program: Less healthy persons tend to choose high-paying, wide-coverage basic medical insurance available for urban employees. Using an instrumental variable to control for the problem of endogeneity, it is discovered that compared with the basic medical insurance system for urban residents, the system for urban employees significantly enhances the health of the senior floating population. Third, “adverse selection” could be observed in the choice between enrolling in health insurance at the place of settlement or another place. Senior migrants with worse self-rated health tend to choose place of settlement in order to enjoy higher compensation and less complex reimbursement procedures. With an instrumental variable to control for the problem of endogeneity, it was found that compared with joining the medical insurance system at other places, joining at a place of settlement could improve the health of the floating senior population.

## 1. Introduction

Since major economic reforms commenced, the health insurance system in China has undergone a process of gradual development. In 1998, the Chinese government established the Basic Medical Insurance System for Urban Employees (BMISUE). This insurance program is in line with the shift to a market-oriented economy and the reform of state-owned enterprises in China. It is a combination of individual contributions and government subsidies. A province, a prefecture-level city, or a county-level city can be the basic pooling district. The insurance fee is proportionally divided among employers and employees [1].

In 2003, in order to solve social security problems in rural areas and improve the health conditions of rural residents (who make up more than 50% of the Chinese population), the Chinese government established the New Rural Cooperative Medical System (NRCMS) for all rural residents. This program follows the principle of voluntary participation. The county or county-level city is the basic pooling unit. The insurance fee is collected by combining individual payments with government subsidies. Both central and local governments allocate special funds to subsidize the program each year. The central government also allows local governments to set different standards for individual payment and government subsidies based on local economic levels.

In 2007, in order to solve the social security problem in urban areas, the Chinese government launched the Basic Medical Insurance System for Urban Residents (BMISUR). This program is designed for self-employed urban residents who do not participate in the basic health insurance system for urban enterprise employees, unemployed urban residents who have reached the legal working age, children, primary and secondary school students, college students, and rural migrants and their children. This program also follows the principle of voluntary participation and combines individual payments with government subsidies. As in the case of NRCMS, both central and local governments allocate special funds to subsidize the program each year, and the standards for individual payment and government subsidies are also different based on different local economic levels.

In 2015, the Chinese government decided to consolidate BMISUR and NRCMS to establish the Basic Medical Insurance System for Urban and Rural Residents (BMISURR). The new BMISURR covers all citizens previously enrolled in BMISUR or NRCMS. This system continues to follow the principle of voluntary participation and combines individual payments with government subsidies. In areas where there is a large gap between the existing payment standards for BMISUR and NRCMS, the local government is allowed to maintain different payment standards for up to three years. At present, this system is gradually being promoted nationwide. Since 2017, more than 50% of the provinces have consolidated the two systems. In brief, China has established a health insurance system for all residents. According to data released by the Chinese government in 2014, governments at all levels invested about 3 trillion yuan to ensure the implementation of national health insurance from 2009 to 2014.

## 2. Literature Review

### 2.1. Impacts of the Health Insurance System on Individual Health

Previous studies showed that health insurance affects individual health status both directly and indirectly. Directly, the financial support provided by health insurance may affect an individual’s psychological stress level [2], which directly affects the individual’s health status. There are three main ways in which health insurance may affect health status indirectly. First, because health insurance provides access to medical care, regular physical examinations, prophylactic treatment, and higher-quality health services, it may contribute positively to an individual’s health [3,4,5]. Second, health insurance may affect health status by influencing personal behaviors. On the one hand, because being enrolled in a program reduces personal expense for medical services, people may actually continue engaging in unhealthy behaviors such as smoking or drinking [6,7]. On the other hand, since people who are enrolled can enjoy more prophylactic health care, they may reduce or quit such harmful behaviors [8]. Finally, health insurance may indirectly affect health status by reducing the uncertainty one feels about future expenses for medical services, thus affecting savings in medical care [9,10].

Many previous studies also focused on correlations between health indicators in people in different age groups from different regions and the insurance programs they enroll in. Most of these studies found that participation in health insurance was significantly associated with lower mortality (or disease risk) and higher self-rated health [11,12]. At the same time, however, some studies indicated no significant correlation between them [13]. Although most of these studies used multivariate regression models to estimate correlations, they had little or no consideration for the interactive relationship between health care and health. They also did not find a causal relationship between the two. Thus, the significance of these studies is limited.

Studies of the causal relationship between health insurance and health are mainly conducted by means of natural experiments. A considerable amount of such literature is focused on analyzing the US health insurance system. Medicare is free health insurance provided by the US government to citizens over the age of 65. Finkelstein and Mcknight (2008) [14] found that Medicare did not significantly reduce mortality in people over 65 during the first 10 years of enrollment. Card, Dobkin, and Maestas (2009) [15] used a regression discontinuity design to study the effects of Medicare on mortality of inpatients over age 65 in the emergency department within seven days. They found that Medicare reduced mortality by 20%.

Medicaid is the general term for health insurance provided by the US government to low-income families. The forms of Medicaid vary in different states, but it is usually provided by the government for free. In 1982, the government of California passed a bill that narrowed the range of application of Medicaid in order to cut down on public expense. Lurie et al. (1986) [16] took this as an opportunity for a natural experiment to compare the health status of two groups of people one year after the bill took effect. One group of people remained enrolled in the program and the other group ceased to be enrolled. The results showed that the average blood pressure of the Medicaid group was higher, but there was no significant difference between the two groups regarding self-reported health status. Currie and Gruber (1996) [17] studied Medicaid as its range of application expanded from 1984 to 1992, using the proportion of children enrolled in each state as the instrumental variable (IV). The results showed that child mortality was reduced by 5.1%. Another study conducted by Goodman showed that the expansion of Medicaid reduced infant mortality and the occurrence of low birth weight [18].

Finkelstein et al. (2012) [4] studied the impact of the Oregon Health Plan (OHP) Standard on the health status of individuals ages 19 to 64. OHP Standard is a form of Medicaid in Oregon that is provided free of charge to low-income citizens of the state. Because the number of applicants exceeds what the project budget can afford, the state government determines who is ultimately eligible to join the program by random selection. Utilizing this special enrollment policy, the study used two-stage least squares (2SLS) to estimate the impact of Standard OHP on health status. They defined the random number assigned by the government as the IV. The results showed that OHP Standard significantly improved self-reported physical and mental health status.

In addition to research in the United States, Hanratty (1996) [19] studied the impact of Canadian universal health care on infant health. From 1962 to 1972, Canada gradually implemented its universal health insurance system nationwide. Hanratty took this gradual process of implementation as an opportunity for a natural experiment and found that universal health insurance reduced infant mortality by 4% and low birth weight by 1.3%. King et al. (2009) [20] studied the impact of universal health insurance in Mexico on the health of the entire population. Aggarwal (2010) [21] used the trend score matching method to examine community-based health care programs implemented in the Yeshavini area of India and found that community health insurance positively affected the health of the enrolled residents by increasing utilization of public medical resources.

### 2.2. Impacts of Health Insurance System on Individual Health in China

Regarding the impacts of the health insurance system in China, several scholars took advantage of the fact that the NRCMS was gradually implemented in different regions to study its impact on the health of rural residents. Two studies [22,23] found that the effect was not significant on the health of rural residents, but Jiang, You, Li, Wei, and Mainstone (2018) [24] found new evidence that NRCMS improved the health status of rural residents. Bai and Wu (2014) [25] analyzed the impact of NRCMS on the nutritional status of the insured elderly based on CLHLS data. Although NRCMS did not significantly improve the dietary diversity of the elderly, researchers found that it did promote the consumption of meat and fish and played a role in the balance of diet and improving the health status of the elders enrolled. The effect was more obvious among men and poor elders. Xie et al. (2018) [26] used panel data of 2000 and 2014 in Jiangxi Province to empirically analyze the impact of NRCMS on the health status of the rural middle-aged and elderly population. The results showed that the implementation of NRCMS had an obvious impact on hospitalization expenses. Wang, Yip, Zhang, and Hsiao (2010) [27] adopted the experimental research method to compare areas where NRCMS had already been implemented with areas where it was yet to be implemented, using random sampling. They found that NRCMS significantly improved the health status of rural residents. This included reducing the rate of self-reported pain and anxiety among rural residents and improving the operational ability of individuals over 55 years of age.

Some studies focused on the impact of BMISUR on the health of urban residents. Luo (2008) [28] used data of the 2012 Chinese Household Income Project (CHIP) survey to study factors influencing the health status of urban residents and patterns of their medical expenses. The results showed that implementation of the insurance system had a significantly negative impact on the health status of urban residents and no significant influence on their self-reported health. Lou ascribed this result to endogenous independent variables. Hu and Liu (2012) [29] utilized propensity score matching and difference-in-difference methods to evaluate the effect of BMISUR. They pointed out that BMISUR improved the health status of low-income sub-healthy urban residents. Pan et al. (2013) [30] also analyzed the impact of BMISUR on the health status of urban residents. They found that participating in BMISUR improved the utilization of urban medical resources and did not cause a financial burden for urban residents. It had a greater impact on people of lower socioeconomic status.

Some studies have focused on the impact of BMISUE on the health of urban residents. Chen and Deng (2016) [31] used data from the 2009 China Health Nutrition Survey (CHNS) to empirically analyze the impact of BMISUE on both the short-term and long-term health status of individuals enrolled. The results showed that BMISUE improved the short-term health status of participants to a certain extent while significantly improving their long-term health. Specifically, it reduced the prevalence of cardiovascular and cerebrovascular diseases. At the same time, participating in BMISUE increased the actual medical expenses of the individuals enrolled.

Other studies have paid particular attention to the impact of the health insurance system on the urban elderly and children. For example, two studies [32,33] focused on the impact of health insurance on mortality among urban elderly. Huang and Wu (2009) [33] identified the average enrolled rate by province, gender, age, and education as the IV, and found that enrolling in a health insurance program reduced the three-year interval mortality rate among the elderly by 25.3%. Under the assumption that insurance status is an exogenous variable, Huang and Gan (2010) [32] found that the risk of death for the elderly enrolled in an insurance system was reduced by 19% compared with those who did not enroll. B. Li and Hu (2010) [34] studied the impact of health insurance on the health of the elderly, using the average community enrollment rate as the IV. They did not find the impact to be significant. Liu, Meng, and Han (2016) [35] used data from the 2006, 2009, and 2011 CHNSs to evaluate the impact of health insurance on children’s health status using the two-stage least squares method. The results showed that health insurance could improve children’s health. Mou and Zhou (2017) [36] used the same data and applied indicators such as health status in the past four weeks, self-rated health status, and body mass index (BMI) to study the impact of NRCMS and BMISUR on children. They found that neither NRCMS nor BMISUR significantly improved the short-term health of children, but NRCUMS significantly reduced the risk of obesity among children. Li and Fang (2018) [37] used data of the 2012 and 2014 China Family Panel Surveys (CFPSs) to analyze the impact of health insurance on children’s health and found that social health insurance significantly improved the self-rated health of children. Enrollment in an insurance program also increased the utilization of medical resources, measured by the number of clinical visits in the past month.

Based on the existing literature, it can be seen that scholars widely agree that the fragmentation in social health insurance schemes in China has produced variations between different groups in terms of utilizing medical services and health status [1]. There are three problems related to the impact of China’s medical insurance system on residents’ health. First, because this insurance program is aimed at a specific group of people, most of the existing research was focused on the impact of a certain insurance program on the health status of its target population. These studies did not give specific attention to the possibly different impacts of different programs. For example, there is no research specifically dedicated to analyzing whether there is a difference in impact of the two main insurance programs in China (BMISUE and BMISURR). Second, the current Chinese health insurance system has not achieved central planning. In some regions, the basic pooling unit is prefecture-level cities and in other regions it is county-level cities. At present, China has 333 prefecture-level administrative divisions (excluding Hong Kong, Macao, and Taiwan) and 2856 county-level administrative divisions (excluding Hong Kong, Macao, and Taiwan). When the place where people enroll in a program is different from the place where they seek medical help, the rates of reimbursement may also be different, thus affecting the utilization of medical resources and the health status of individuals enrolled. Third, in the studies of the health status of specific groups of people, such as the elderly population and children, no research has been conducted to analyze the impact of the health insurance system on the health status of the floating population.

This article has focused on senior floating population based on the three following reasons. First, because of rapid urbanization in recent years, the size of the floating population in China has grown at an alarming rate. According to data released by the Chinese government, the total number of migrants in China continually increased from 230 million in 2011 to 245 million in 2017. The floating population was made up of 17.72% of Chinese in 2017. As a constituent part of China’s floating population, senior floating population’s health at place of settlement and whether migration has affected their health deserves scholarly attention. Second, from a medical or physiological perspective, the senior population has a higher risk of various disease than younger populations, so examining the senior floating population’s health status and its relationship with insurance enrollment may yield more typical results. Third, more importantly, the current Chinese health insurance system offers multiple combinations of insurance programs and place of enrollment, providing an opportunity to compare the effects of different insurance programs on individual health.

In China, insurance programs are designed for specific social groups, such as urban employees, urban residents and rural residents, so that people of each social group can enroll in insurance programs according to their social identity. Yet the senior floating population, as a special social group that moves between different regions and between different occupations, are qualified to enroll in more than one kind of insurance program. This offers us an opportunity to compare the effects of different insurance programs and of the same program in different regions.

Under the current health insurance system, where prefecture-level and county-level cities serve as the basic pooling units, there are five possibilities for the floating population: (1) Enroll in BMISUE at the place of settlement. (2) Enroll in BMISURR at the place of settlement. (3) Enroll in BMISUE at a place other than the place of settlement. (4) Enroll in BMISURR at a place other than the place of settlement. (5) Do not enroll in any insurance program. ‘Place of settlement’ in this article refers to the city to which the senior migrants have immigrated, where they presently live and work.

It should be noted that, as mentioned in the introduction, since 2015, some regions have started to consolidate BMISUR and NRCMS to form the new BMISURR. Thus, the distinction between NRCMS and BMISUR was no longer applicable in 2015 when the data were collected. Therefore, in this paper, when the respondents report themselves as enrolling in BMISUR, NRCMS, or BMISURR, their responses are regarded as enrolling in BMISURR. That is to say, enrollment in BMISURR, as defined in this paper, includes enrollment in BMISUR, NRCMS, or BMISURR. This paper uses 2015 China Migrants Dynamic Survey data collected by the National Health Commission to study the health status of the elderly migrant population and analyzes the impact of place of enrollment within the Chinese health insurance system. The floating population studied in this paper is defined as those whose current place of work and residence is different from their place of residence registration (or “Hukou”). Under the special registration system currently implemented in China, each citizen is registered in the place where he or she was born (this can be a city, a prefecture or a county). When a citizen migrates to other places, he or she can either change their place of residence registration to their new place of living or remain to be registered in their place of birth. However, in practice, because of the complexity and triviality of the bureaucrat procedures, most migrants, especially seniors, will not change their Hukou after migrating. In this study, the “floating population” is defined as a person who lives in a place of residence for one month or longer without a local residence registration.

## 3. Methods

### 3.1. Data

We used the 2015 China Migrants Dynamic Survey (CMDS), which is based on the annual nationwide survey of the migrant population conducted by the National Health Commission of PRC since 2009. This survey has a sample size of around 200,000, drawn from 31 provinces (autonomous regions and municipalities). In 2015, CMDS drew the sample from male and female migrants ages 15 and above who were not registered in the local area (city or county) for 1 month or more. The sampling frame is the 2014 annual reports of the floating population of 31 provinces (autonomous regions/municipalities). The stratified multistage probability proportional to size (PPS) method was used for sampling. During the first stage, townships/subdistricts were selected according to the PPS method. During the second stage, village/community groups were selected from these townships/subdistricts according to the PPS method. At the third stage, individual participants were randomly sampled from these village/community groups. The 2015 CMDS focuses on the life and development of the floating population and the utilization of public health services by seniors (defined as people 60 years of age and above, determined by birth date on the questionnaire before May 1955). The original data consists of 206,000 samples in total, including 4484 seniors. This paper analyzes these 4484 seniors to examine the impact of different health insurance programs on the health of the senior floating population.

### 3.2. Variables

Dependent Variable. In this study, “self-rated health” is used to measure the health status of senior migrants, with values 1–4 representing “very unhealthy,” “unhealthy,” “basically healthy,” and “very healthy.” Table 1 shows the distribution of self-rated health values of senior migrants. It can be seen that the proportion of “very healthy” is the highest (45.94%), followed by “basically healthy” (44.45%). The proportion of “very unhealthy” seniors is the lowest (0.91%).

Independent Variables. As mentioned above, China’s health insurance system takes cities and counties as pooling districts. Consequently, enrolling in different programs and applying at different places can affect the way migrants utilize public medical resources, and might influence their health as a result. Therefore, in this paper, we distinguish between different health insurance programs and the places where one applies for health insurance. In empirical research, according to the program and the place of application, we separated senior migrant participants in the medical insurance system into f5 categories: BMISURR (place of settlement), BMISURR (other place), BMISUE (place of settlement), and BMISUE (other place) and Unknown or not participating in health insurance system. It should be noted that BMISURR represents 3 possible situations: BMISURR, NRCMS, or BMISUR. The combination of NRCMS and BMISUR was officially implemented in 2016, but many local governments have been experimenting with the combination since 2015, which explains why there are 3 possible answers. In this paper, we compress them into one category, BMISURR. Table 2 shows the medical insurance coverage of our senior floating population sample. Most notably, more than half fall into the category BMISURR (other place) (50.71%), while the proportion of BMISUE (place of settlement) is the lowest, only 2.19%. In addition, 16.46% of these senior migrants did not report participating in the health insurance system.

Controlled Variables. In addition to medical insurance participation, in this research we also controlled for individual characteristics of the senior floating population, i.e., gender, age, ethnicity, household registration type, marital status, education level, and monthly household income. Other variables that may influence individual health are controlled for as well, i.e., range of migration, main source of income, number of friends at the place of settlement, daily exercise time, and physical examinations taken. Finally, considering that the economic and social development of provinces and municipalities in China vary greatly, and that different provinces and municipalities adopt different health insurance policies, we tried to minimize their influence by controlling for place of settlement. The definitions of controlled variables and a description of the mean are shown in Table 3.

### 3.3. Measurement Model

Since the dependent variable is an ordinal variable of 1–4, we use the ordered probit model to estimate the impact of health insurance on health. The basic model is:

$$Y_{i}=\beta_{0+}\beta_{1}{\mathrm{Insurance}}_{i}+\beta_{2}{\mathrm{Person}}_{i}+\beta_{3}{\mathrm{Control}}_{i}+\varepsilon_{i}$$

where $Y_{i}$ represents the individual’s health, $\beta_{0}$ the intercept, and $\beta_{1}$, $\beta_{2}$, and $\beta_{3}$ the coefficients affecting the individual’s health. $\beta_{1}$ represents the coefficient of health insurance’s impact on health. ${\mathrm{Insurance}}_{i}$ indicates the individual’s participation in health insurance. ${\mathrm{Person}}_{i}$ indicates a series of personal characteristics, i.e., gender, age, ethnicity, household registration type, marital status, and education level. ${\mathrm{Control}}_{i}$ indicates other control variables, i.e., range of migration, monthly household income, main source of income, number of friends, daily exercise time, physical examination, and inflowing provinces $\varepsilon_{i}$ is a random disturbance term.

## 4. Results

### 4.1. The Ordered Probit Regression Results of the Effect of Health Insurance on the Health of China’s Senior Migrants

Table 4 shows the results of the ordered probit regression of health insurance regarding the health of migrants in China. In Model 1, the senior floating population is divided into two categories: Those who have enrolled in health insurance and those who have not (variable Y “insurance” indicates enrollment in health insurance). It is shown that, under the same conditions, seniors who have enrolled in health insurance are more likely to be healthy.

For the purpose of identifying the impact of different types of health insurance on the health status of the senior floating population, Model 2 categorizes health insurance for this population into three types: medical insurance for rural and urban residents (BMISURR), medical insurance for urban employees (BMISUE), and no health insurance. The baseline variable is no health insurance. As the results show, under the same conditions, compared with senior migrants without medical insurance, the health status of senior migrants who have BMISURR is significantly improved, while those who have BMISUE do not have significant improvement in health status.

In order to further analyze the impact of different places of medical insurance enrollment on the health of migrants, Model 3 categorizes senior migrants’ health insurance enrollment according to place of enrollment into five groups: BMISURR (settlement), BMISURR (nonsettlement), BMISUE (settlement), BMISUE (nonsettlement), and no health insurance. The baseline variable is no health insurance. The results show that compared with not having health insurance, enrolling in BMISURR and BMISUE at places other than the place of settlement could result in significantly higher health status, but enrolling in BMISURR or BMISUE at the place of settlement has no significant effect on the health of senior migrants.

At the same time, considering that people with poor health may reduce the times of physical exercise due to physical reasons, may be more likely to take physical examinations, and may also have decreased income and less interaction with friends, in this article various endogenous controls are excluded including the number of friends at place of settlement, daily exercise time, physical examination results of the past year and family income. After re-estimation, the results show no significant changes (The coefficient of the variable ‘insurance’ from Model 1 becomes 0.117, significant at the level of 10%. The coefficient of the variable BMISURR in Model 2 becomes 0.109, significant at the level of 10%; the coefficient of the variable BMISUE becomes 0.103, not statistically significant. The coefficient of the variable BMISURR(Settlement) in Model 3 becomes 0.0421, not statistically significant; the coefficient of the variable BMISURR (Nonsettlement) becomes 0.120, significant at the level of 5%; the coefficient of the variable BMISUE (Settlement) becomes −0.0177, not statistically significant; the coefficient of the variable BMISUE (Nonsettlement) becomes 0.165, statistically significant at 10%).Regression results show that other conditions being the same, (1) the health status of male senior migrants is significantly higher than that of female senior migrants; (2) senior migrants who are older report worse self-rated health status than younger ones; (3) senior migrants who have higher education levels report better self-rated health status than those with less education; (4) daily exercise time, monthly household income, and whether one has physical examinations are all positively correlated with self-rated health of senior migrants; (5) compared with those who receive their income mainly from other family members, those with employment or pension/savings as their main source of income report better self-rated health; and (6) compared with seniors who migrated within a city or from one county to another, the health of those who migrated across provinces show better health status, which may be because senior migrants who are in good health are more willing to travel over long distances.

### 4.2. The IV Regression Results of the Effects of Health Insurance System on the Health of China’s Senior Floating Population

#### 4.2.1. The IV Regression Results of the Effects of Different Health Insurance System on the Health of China’s Senior Floating Population

Because there is remarkable difference between BMISURR and BMISUE in terms of fee payment and insurance rate, there can be adverse selection during the process of senior floating population selecting health insurance under the influence of varying health conditions. When there is adverse selection in the choice of insurance programs by the senior floating population, ordered probit analysis is supposed to have endogenous problems, resulting in biased estimation of the parameters. This paper therefore uses instrumental variable (IV) to identify the causal effects of health insurance on health. Drawing on the research of Pan et al. (2013) [30], this research takes the proportion of government subsidies to total funding of BMISURR in respective cities in 2015 as the IV. As mentioned in the introduction section, the main source of funding of China’s BMISURR is individual payment and government subsidy. In 2015 CMDS data, the places of settlement of senior floating population includes 301 cities of China. The city with lowest ratio of government subsidy has a subsidy ratio of 65.21%, while the city with the highest has a subsidy ratio of 88.09%, averaging 81.13%. It should be noted that when local governments separately collect the information of the government subsidy of NRCMS and BMISUR, BMISURR is calculated as the sum of both insurance programs.

An instrumental variable must satisfy two conditions: First, it must be significantly correlated with the endogenous variable. Thus, it has to be determined whether the government subsidy ratio is highly correlated with the type of medical insurance selected by the individual. It can be reasonably assumed that the greater the proportion of government subsidies for BMISURR, the smaller the premium paid by each individual and the greater the willingness of individuals to enroll. The subsidy ratio could therefore be highly correlated with an individual’s choice of insurance program. Second, the instrumental variable must be exogenous. The subsidy ratio can only affect the individual choice of insurance programs, and there can be no other process through which it affects individual health status. It can be safely assumed as exogenous. Other studies have similarly used regional insurance policies as instrumental variables for individual enrollment [38,39].

Table 5 removes 738 individuals with no medical insurance and reports the IV regression results of the relationship between different health insurance systems and health. The regression results of Stage I show that government subsidy ratio for BMISURR in 2015 is significantly negatively related to senior migrants’ participation in BMISUE, or in other words, for senior floating population participating in medical insurance, if the government of place of settlement gives a higher subsidy ratio to BMISURR, the senior floating population will be more inclined to participate in BMISURR, and less inclined to participate in BMISUE. The method of Staiger and Stock (1997) [40] for testing weak instrumental variables is used on this IV, producing an F-value exceeding the critical value for rejecting IV of around 16 on the level of 10%, which indicates that this IV passed the test [41].

As Stage II of Table 5 indicates, after adjusting for the endogenous problem, it is found that compared with BMISURR, enrolling in BMISUE has a positive impact on the health of the senior floating population. This supports the hypothesis that BMISUE can increase participants’ utilization of medical services, thereby improving their health.

#### 4.2.2. IV Regression Results of the Effect of Place of Enrollment on the Health of the Senior Floating Population

As mentioned above, in China, basic medical insurance systems do not take the whole country as a pooling unit, but prefecture- and city-level administrative units. If the place of enrollment and place of hospital visits happen to be in the same pooling unit, the reimbursement rate will be relatively high and the reimbursement procedure is convenient. Therefore, enrolling in medical insurance at the place of settlement could better facilitate utilization of medical services, thereby promoting the health of the senior floating population.

Table 6 analyzes the causal relationship between different regions of insurance enrollment in BMISURR and the health of senior migrants, with the 2634 senior migrants who have enrolled in BMISURR as sample. The Stage I regression results in Table 6 show that in 2015, the government’s subsidy ratio for BMISURR in place of settlement was significantly positively related to the participation of the senior floating population in BMISURR in place of settlement. That is to say, the higher the government subsidy ratio for BMISURR, the more willing the senior floating population is to enroll in BMISURR in their place of settlement. The method of Staiger and Stock (1997) [40] for testing weak instrumental variables is used on this IV, producing an F-value of 25.69, exceeding the critical value for rejecting IV of around 16 on the level of 10%, which indicates that this IV passed the test [41]. It is also reasonable to believe that the government subsidy ratio of BMISURR at place of settlement could affect individual health only through affecting individual choice to enroll in BMISURR in place of settlement or not, and there is no other possible influencing process.

## 5. Discussion

### 5.1. Health Insurance Programs and the Health of the Senior Floating Population

The regression results of Model 1 in Table 4 show that enrolling in health insurance is significantly correlated with better self-rated health of senior migrants. As the studies of Finkelstein et al. (2012) [4] and Card et al. (2009) [15] explained, health insurance may benefit the health of participants by improving the financial accessibility of medical services, thus encouraging their utilization and promoting health.

The regression results of Model 2 in Table 4 found that, compared with senior migrants without health insurance, senior migrants with BMISURR reported significantly better health, while those with BMISUE reported no significant difference in health status. Regression results of Stage II of Table 5 show that compared with senior migrants enrolling in BMISURR, enrolling in BMISUE has a positive influence on senior migrants’ health, indicating that the relatively high level of compensation of BMISUE has a positive effect on senior migrants’ health.

The difference between Table 4 and Table 5 implies that when senior migrants enroll in medical insurance, a situation of adverse selection occurs, namely that healthy people tend to choose the low-paying, low-reimbursing BMISURR, while those with poor health tend to choose the high-paying, high-reimbursing BMISUE. Adverse selection is a common phenomenon in the insurance market. Akerlof (1970) [42] discovered this phenomenon in the commercial insurance market and explained that because of information asymmetry, policyholders can maximize their profit by selecting the insurance program that is most profitable for them, resulting in low insurance efficiency and quality. Similarly, in the health insurance market, information asymmetry also exists, and senior migrants can choose the program that is most beneficial for them.

According to statistics of the National Bureau of Statistics of China in 2015, the average premium for BMISUE was 4223 yuan, while the average premium for BMISURR was 545 yuan (with an average government subsidy of 410 yuan). As for reimbursement, BMISUE has a higher coinsurance rate, more counties capable of reimbursing outpatient expenses or catastrophic/chronic disease expenses, and a higher upper limit for reimbursement [43]. In brief, BMISUE is high-paying, high-reimbursing insurance, while BMISURR is low-paying, low-reimbursing insurance. Results have shown that the less healthy senior floating population tend to choose the high-paying, high-reimbursing BMISUE, in order to maximize their benefits, while those with better health and expect lower chances of utilizing public medical resources tend to choose the low-paying, low-reimbursing BMISURR (average individual payment of BMISURR in 2015 is 135 yuan). Therefore, it can be seen that BMISUE significantly improves the health of senior migrants enrolling in it is because it has a high reimbursement rate, despite high individual payment. When senior migrants in BMISUE are diseased, considering that BMISUE has a much higher upper limit for in-patient and hospitalization service, they are more likely to go to the hospital for medical services. Thus, enrolling in BMISUE will increase the utilization of medical resources for senior migrants, and benefit their health.

### 5.2. Place of Enrollment and the Health of the Senior Floating Population

In Model 3 of Table 4, in order to analyze the impact of different places of enrollment on the health of senior migrants, we further subdivide the medical insurance programs in which senior migrants have enrolled into: BMISURR (place of settlement), BMISURR (other place), BMISUE (place of settlement), BMISUE (other place). The regression results of Model 3 show that, compared with not having health insurance, enrolling in either insurance program at a place other than the place of settlement has a positive correlation with the health of senior migrants. Enrolling at the place of settlement, on the other hand, whether in BMISUE or BMISURR, has no significant impact on their health.

From the Stage II regression results of Table 6, it can be seen that compared with those who enrolled in BMISURR at other places, those who enrolled in BMISURR at the place of settlement report better health. The difference between Table 4 and Table 6 implies that when senior migrants enroll in medical insurance at different places, a situation of adverse selection occurs, namely that less healthy people tend to choose to enroll in BMISURR at place of settlement, while those with good health tend to choose to enroll in BMISURR at other places (mainly at place of household registration).The main reason that adverse selection occurs is that, a possible explanation is that in China, basic medical insurance systems do not take the whole country as a pooling unit, but prefecture- and city-level administrative units. If the place of enrollment and place of hospital visits happen to be in the same pooling unit, the reimbursement rate will be relatively high and the reimbursement procedure is convenient. On the other hand, if the place of enrollment and place of hospital visits are not in the same pooling unit, the reimbursement rate for medical expenses will be relatively low and patients will be required to acquire medical referral certificates and invoices for medical visits from the hospital and return to their place of enrollment to complete the reimbursement process, which can be inconvenient for many.

For example, according to the stipulation of the administrative department of Henan Province of China concerning the proportion of reimbursement for BMISURR, if one is enrolled in a municipal-level administrative area within Henan province and attending a hospital in the place of enrollment, the deductible line for reimbursement is 500 yuan, and the reimbursement rate is 55% (for 500–3000 yuan) and 75% (for over 3000 yuan); if they visit a provincial hospital outside the place of enrollment, the deductible line for reimbursement will be raised to 600 yuan, and the reimbursement rate will be reduced to 53% (for 600–4000 yuan) and 72% (for over 7000 yuan); if they go to a hospital outside Henan province, the deductible line will be raised to 1500 yuan, and the reimbursement rate will be reduced to 50% (for 1500–7000 yuan) and 68% (for over 7000 yuan).

For China’s senior floating population, especially those who undergo frequent migration, few of them have the ability or intention to settle for the long term in their current place of residence. Under the influence of Chinese traditions, many of them would prefer to return to their place of birth in the future. Therefore, when senior migrants are in good health, they tend to enroll in BMISURR at place of household registration, while for those with poor health and high risk of immediate hospitalization, in order to have more convenient medicalization procedures and higher reimbursement, they would choose to enroll in BMISURR at place of settlement. The main reason why enrolling in BMISURR at the place of settlement significantly improves senior migrants’ health is that senior migrants who enroll in BMISURR at the place of settlement have higher reimbursement rates and more convenient procedures, thus promoting utilization of medical services and improving their health.

## 6. Conclusions

This paper analyzes the impact of health insurance on the health of the senior floating population in China using data of the 2015 China Migrants Dynamic Survey. Taking into consideration that the senior floating population will enroll in different medical insurance programs according to their own health conditions, the government subsidy ratio is used as an instrumental variable to pinpoint the impact of different medical insurance programs on the health of the senior floating population. We have drawn the following conclusions: First, enrolling in medical insurance significantly improves the self-rated health of the senior floating population, which confirms the findings of other studies on this topic, such as those of Huang and Gan (2010) [32], that seniors who enroll in medical insurance will increase their use of medical services to improve their health. Second, senior migrants’ choice of health insurance program is influenced by their health status. Senior migrants with poor health are more inclined to choose the high-paying, high-reimbursement BMISUE. Using the instrumental variable to control for the endogenous problem, we found that BMISUE significantly improved the health of the senior floating population compared with BMISURR. Third, 58.76% of our sample seniors have enrolled in BMISURR. Whether they chose to enroll in the insurance program at their place of settlement or another place, the phenomenon of adverse selection could be observed. That is, senior migrants with poor health status tend to enroll in BMISURR at their place of settlement. This might be explained by the fact that enrollment at the place of settlement could bring higher reimbursement rates and more convenient procedures relative to enrollment at other places. When using the instrumental variable to control for endogenous problems, we found that enrolling in BMISURR at the place of settlement could better improve the health of senior migrants compared with enrollment at other places.

## 7. Limitation

It should be noted that this study still has some limitations. First, self-rated health is used by the survey as the only measurement of health status, which does not allow us to examine health status through other objective measurements. Second, we failed to find a suitable instrumental variable to conduct an analysis of causality concerning place of enrollment in BMISUE and the health status of its members. Therefore, when identifying the impact of place of enrollment on individual health, only BMISURR is taken as an example. Third, the senior floating population, that is, the object of study of this article, is a special subgroup of the senior population in China. Poor health conditions and cultural preferences of the elderly to stay in a familiar place and lead a peaceful life could reduce senior population’s desire to migrate. Therefore, the conclusions reached by this article could be generalized to China’s senior floating population, but not to the entirety of China’s senior population. Future studies are expected to expand the object of study to China’s senior population. 

## Figures and Tables

**Table 1:** Self-rated health value sampling distribution of the senior floating population.

Self-Rated Health	Very Healthy	Basically Healthy	Unhealthy	Very Unhealthy	Total
No.	2060	1993	390	41	4484
Proportion	45.94%	44.45%	8.70%	0.91%	100%

**Table 2:** Sample distribution of health insurance participation of senior floating population of China. BMISURR, Basic Medical Insurance System for Urban and Rural Residents; BMISUE, Basic Medical Insurance System for Urban Employees.

Health Insurance	BMISURR (Place of Settlement)	BMISURR (Other Place)	BMISUE (Place of Settlement)	BMISUE (Other Place)	Unknown or Not Participating in Health INSURANCE System	Total
No.	360	2274	98	1014	738	4484
Proportion	8.03%	50.71%	2.19%	22.61%	16.46%	100%

**Table 3:** Definitions of controlled variables and description of the mean.

Variable		Definition	Mean	Std. Dev.
	Gender	Dummy variable: male = 1, female = 0	0.6075	0.4884
	Age	Continuous variable: age of the participant	66.2507	5.6929
	Ethnicity	Dummy variable: Han ethnicity = 1, other ethnic minorities = 0	0.9086	0.2883
Type of Household Registration (Hukou)	Rural	Dummy variable: rural hukou = 1, others = 0	0.6010	0.4897
	Nonrural	Dummy variable: nonrural hukou = 1, others = 0	0.3805	0.4856
	Rural to Resident	Dummy variable: rural to resident hukou = 1, others = 0	0.0123	0.1101
	Nonrural to Resident	Dummy variable: nonrural to resident hukou = 1, others = 0	0.0062	0.0788
Marital Status	Single	Dummy variable: single = 1, others = 0	0.0091	0.0952
	Married	Dummy variable: married = 1, others = 0	0.8062	0.3953
	Divorced/Widowed	Dummy variable: divorced/widowed = 1, others = 0	0.1847	0.3881
	Education Level	Categorical variable: unschooled = 1, primary school = 2, middle school = 3, high school or technical secondary school = 4, postsecondary college = 5, university = 6, postgraduate = 7	2.6062	1.1101
Range of Migration	Interprovince	Dummy variable: participant migrated from one province to another = 1, otherwise = 0	0.4204	0.4937
	Intercity	Dummy variable: participant migrated from one city to another within the same province = 1, otherwise = 0	0.3147	0.4644
	Intercounty	Dummy variable: participant migrated from one county to another within the same province = 1, otherwise = 0	0.2649	0.4414
	Monthly Household Income	Continuous variable: monthly household income of the participant (after tax)	4975.873	4596.587
Main Source of Income	Employment	Dummy variable: participant’s main source of income is his/her own employment = 1, otherwise = 0	0.3073	0.4614
	Pension/Savings	Dummy variable: participant’s main source of income is pension or savings = 1, otherwise = 0	0.4222	0.4940
	Other Family Members	Dummy variable: participant’s main source of income is from other family members = 1, otherwise = 0	0.2248	0.4175
	Others	Dummy variable: participant’s main source of income is from none of the three aforementioned categories = 1, otherwise = 0	0.0457	0.2089
	No. of Friends at Place of Settlement	Continuous variable: no. of friends at place of settlement	8.4683	10.8908
	Daily Exercise Time (min)	Continuous variable: average time spent exercising daily, in minutes	66.0203	45.2182
	Physical Examination	Dummy variable: participant has had at least one physical examination in the past year = 1, otherwise = 0	0.3562	0.4789

**Table 4:** Ordered probit regression results of the impact of health insurance on China’s senior floating population.

Variables		Model 1	Model 2	Model 3
		Coef./Std.Err.	Coef./Std.Err.	Coef./Std.Err.
	Health insurance	0.0910 *		
		(0.0469)		
	BMISURR		0.0887 *	
			(0.0487)	
	BMISUE		0.0990	
			(0.0643)	
BMISURR	Settlement			0.00900
				(0.0784)
	Nonsettlement			0.102 **
				(0.0496)
BMISUE	Settlement			−0.0496
				(0.118)
	Nonsettlement			0.116 *
				(0.0657)
	Gender	0.0798 **	0.0796 **	0.0787 **
		(0.0381)	(0.0381)	(0.0381)
	Age	−0.0396 ***	−0.0396 ***	−0.0397 ***
		(0.00328)	(0.00328)	(0.00328)
	Ethnicity	−0.00866	−0.00872	−0.0165
		(0.0599)	(0.0599)	(0.0602)
Hukou	Rural hukou	−0.0439	−0.0409	−0.0433
		(0.0495)	(0.0518)	(0.0518)
	Rural to resident hukou	0.201	0.204	0.201
		(0.166)	(0.168)	(0.167)
	Nonrural to resident hukou	0.542 **	0.540 **	0.540 **
		(0.219)	(0.220)	(0.219)
Marital Status	Single	−0.145	−0.144	−0.142
		(0.187)	(0.187)	(0.187)
	Married	0.00653	0.00634	0.00442
		(0.0480)	(0.0480)	(0.0482)
	Education level	0.0506 ***	0.0502 ***	0.0498 ***
		(0.0186)	(0.0188)	(0.0188)
Range of Migration	Interprovince	0.179 ***	0.179 ***	0.180 ***
		(0.0462)	(0.0462)	(0.0463)
	Intercity	−0.0514	−0.0515	−0.0518
		(0.0459)	(0.0459)	(0.0460)
Main Source of Income	Income from employment	0.715 ***	0.715 ***	0.715 ***
		(0.0547)	(0.0547)	(0.0547)
	Pension/savings	0.214 ***	0.211 ***	0.207 ***
		(0.0552)	(0.0579)	(0.0580)
	Others	0.164 *	0.164 *	0.168 *
		(0.0983)	(0.0983)	(0.0984)
	Physical examination	0.112 ***	0.112 ***	0.117 ***
		(0.0371)	(0.0371)	(0.0371)
	No. of friends of settlement	0.00268	0.00267	0.00278
		(0.00174)	(0.00174)	(0.00174)
	Daily exercise time	0.00218 ***	0.00218 ***	0.00218 ***
		(0.000433)	(0.000434)	(0.000434)
	Household income	2.09 × 10^−5^ ***	2.09 × 10^−5^ ***	2.05 × 10^−5^ ***
		(6.12 × 10^−6^)	(6.12 × 10^−6^)	(6.06 × 10^−6^)
	Province of settlement	control	control	control
	*N*	4484	4484	4484
	Pseudo-R^2^	0.0800	0.0800	0.0804

Note: The baseline variable in Models 1, 2, and 3 for “enrolled in health insurance” is no medical insurance. The baseline variables for household registration (hukou) type, marital status, range of migration, and main source of income are nonrural hukou, divorced or widowed, intercounty, and other family members. *, **, *** indicate significance at levels of 10%, 5%, and 1%, respectively.

**Table 5:** IV Regression of health insurance and the health of the senior floating population.

Variable	Stage I.	Stage II.
	BMISUE	Self-Rated Health
Government Subsidy Ratio of Place of Settlement in 2015	−0.104 ***	
	0.0247	
BMISUE		0.0125 *
		0.00749
Controlled variables	control	control
Province	-	-
N	3746	3746

Note: Controlled variables for Stage I and Stage II are the same as Table 4, so they are not listed here. In Stage II, the baseline variable for BMISUE is BMISURR. *, *** indicates significance at the level of 10%, 5% and 1%.

**Table 6:** IV Regression of place of enrollment and the health of the senior floating population.

Variable	Stage I.	Stage II.
	BMISURR-Settlement	Self-Rated Health
Government Subsidy Ratio of Place of Settlement in 2015	−0.227 ***	
	0.0405	
BMISURR—settlement		0.0826 *
		0.0497
Controlled variables	control	control
Province	-	-
N	2634	2634

Note: Controlled variables for Stage I and Stage II are the same as Table 4, so they are not listed here. In Stage II, the baseline variable for BMISURR-settlement is “enrolling in BMISURR in other place”. *, *** indicates significance at the level of 10%, 5% and 1%.
